# Refining the diagnosis of 46,XY disorders of sex development: insight from whole-exome sequencing

**DOI:** 10.1186/s13023-026-04453-9

**Published:** 2026-06-27

**Authors:** Ewa Błaszczyk, Małgorzata Więcek, Aleksandra Jazela-Stanek, Grzegorz Kudela, Tomasz Koszutski, Karolina Kowalczyk, Jagoda Sikora, Agnieszka Wiernik, Małgorzata Żarczyńska, Wiktoria Kempińska, Agnieszka Bielska-Brodziak, Aneta Gawlik-Starzyk

**Affiliations:** 1 Departments of Pediatrics and Pediatric Endocrinology, Faculty of Medical Sciences in Katowice, Medical University of Silesia, Katowice, Poland; 2 Department of Genetics and Clinical Immunology, National Institute of Tuberculosis and Lung Diseases, Warsaw, Poland; 3 Department of Pediatric Surgery and Urology, Medical University of Silesia, Katowice, Poland; 4 Chair and Department of Gynecology, Obstetrics and Oncological Gynecology, Medical University of Silesia, Katowice, Poland; 5 Faculty of Law and Administration, University of Silesia, Katowice, Poland

**Keywords:** Differential sexual development, Karyotype 46, XY, Whole-exome sequencing, Variant of uncertain significance

## Abstract

**Introduction:**

Differences in sex development (DSD) with 46,XY karyotype are a group of rare congenital conditions affecting the structure and function of the urogenital system. Published data indicate, that despite the increasingly widespread use of genetic testing, the etiology remains unclear in approximately half of cases.

**Aim of the study:**

To clarify the molecular causes of 46,XY DSD by performing whole-exome sequencing (WES) in a precisely phenotyped and clinically comprehensively evaluated group of patients.

**Patients and methods:**

WES was performed in a consecutive cohort of 39 children diagnosed in our center as 46,XY DSD (aged 0.2–17.9 years). 32 were assigned male, 6 female, and 1 was a transgender boy. All patients underwent detailed clinical, hormonal and biochemical evaluation prior to genetic testing.

**Results:**

A genetic cause explaining DSD phenotype was identified in 8 children. Pathogenic variants were detected in 3 patients, including variants in the *AR* and *DHX37* genes. Likely pathogenic variants were found in 5 patients, affecting the *AR* and *HSD17B3* genes. Variants of uncertain significance (VUSs) were identified in 7 patients, involving genes with well-established relevance to DSD- *NR5A1, DHX37, AR, MAMLD1, SOS2*and*FAM111A*. Although classified as VUSs these variants represent plausible contributors to the patients’ phenotypes. In the remaining children, no variants currently known or suspected to be associated with 46,XY DSD were identified. The most common confirmed etiology in the cohort was androgen insensitivity syndrome (AIS). In addition, pathogenic variants in genes not linked to DSD were identified in 7 patients, demonstrating the broader clinical utility of WES.

**Conclusions:**

Our findings confirm that even a broad, high-throughput method such as WES fails to establish the molecular cause of 46,XY DSD in a substantial proportion of well-phenotyped patients, while at the same time enabling the identification of pathogenic variants in genes unrelated to DSD. We observed frequent genotype–phenotype discordance: similar clinical phenotypes could be associated with different genotypes, whereas the same gene variant could present with variable clinical expression. Re-analysis of WES data after 12–24 months should be considered in patients without a definitive diagnosis or in those who develop additional clinical features.

## Introduction

Differences in sex development (DSD) with a 46,XY karyotype represent a heterogeneous group of conditions characterized by atypical development of chromosomal, gonadal, or anatomical sex. In humans, many genes are involved in sex determination, but the most important and key gene in the development of male sexual characteristics is the *SRY* gene (Sex-determining Region Y), located on the Y chromosome[1–3]. In addition to *SRY*, other genes play roles in sexual differentiation – these are genes responsible for testis determination (*SOX9, NR5A1*), gonadal development or steroidogenesis pathways (*AR, SRD5A2, HSD17B3*) [4, 5]. The exact frequency of individual with DSD 46,XY genetic disorders is difficult to determine due to the heterogeneity of the disorders and different methods of their diagnosis. Defects in androgen receptors and synthesis (*AR, SRD5A2, HSD17B*) collectively can explain the majority of genetically confirmed 46,XY DSD [6], the most common of which is androgen insensitivity syndrome (AIS) [7]. Mutations in gene *NR5A1* are also significant, particularly in cases of gonadal dysgenesis [8] as well as deficit of 5-alfa-reductase enzyme is also the most frequently observed [7]. In worldwide cohort study the most commonly mutated genes associated with androgen synthesis and action were *AR, SR5A2*, and *HSD17B3*, and the most commonly mutated genes involved in gonadal formation were *NR5A1* and *MAP3K1* [5].

Genetic testing has long been a cornerstone in the diagnostic assessment of 46,XY DSD, with a range of methodologies utilised over the years in accordance with technological developments.The primary method is karyotype testing using conventional cytogenetic techniques. Faster molecular cytogenetic techniques that do not require cell culture is an array comparative genomic hybridization (aCGH) that can identify submicroscopic genome imbalance. Detected copy number variation (CNV) can include microdeletions and microduplicatons between 10 kb and 5 Mb in size that may contain a several genes [9]. So these changes can be found in patients with an apparently normal karyotype. The next step in genetic testing should be sequencing. Sanger sequencing may be considered in case of strong suspicion of patient diagnosis being a method of choice in case of *AR* and *SRD5A2* defects [10, 11]. In less obvious cases, next-generation sequencing (NGS) techniques are recommended such as gene panels or whole-exome sequencing (WES). These newer molecular genetic diagnostic strategies are improving diagnostic yield. Further investigation by WES may identify novel genes involved in the development of the phenotype. [12, 13]. Ongoing development of knowledge about embryonic and foetal development may lead to find more candidate genes for DSD.

However, published data show that despite the increasingly frequent genetic diagnostics, the causes remain unclear in about half of cases with 46,XY DSD. Even in large NGS panels or WES, a molecular diagnosis is achieved in only about 31–52% of cases [4, 7, 14]. Thus substantial portion of patients remain without a definitive genetic explanation, even after advanced molecular testing. Contributing factors include incomplete knowledge of causative genes, undetectable regulatory or structural variants, and the presence of variants of uncertain significance (VUS). Due to the continuous development of knowledge in the field of genetics, we conducted our own studies based on the WES method trying to identify the causes of DSD in patients with 46,XY karyotype being under the care of our center.

## Subjects and methods

In a group of 39 patients with confirmed by karyotyping 46,XY DSD (aged 0.2–17.9) diagnosed at the Departments of Pediatrics and Pediatric Endocrinology, WES was performed between March 2023 and March 2025. Male gender was assigned at birth in 32 patients, female gender in 7. One patient currently identifies as a transgender boy.

### Clinical and biochemical phenotype

A detailed description of the clinical and biochemical profile was performed. A physical examination was performed, including assessment of anthropometric measurements, assessment of puberty using the Tanner scale [15], and assessment of masculinization based on the EGS scale [16]. Data was entered in accordance with the medical records from patient visits. Hormonal evaluation and ultrasound tests were performed, and in the case of older patients, a psychological consultation was also conducted, including assessment of gender identity. The results of hormonal tests are presented in Table 1. Each patient has performed evaluation of karyotype using conventional cytogenetic techniques according to the recommendations of the American College of Medical Genetics. The information related to existed co-morbidities was included in phenotype presentation and referral to WES in order to search for their potential genetic causes.

**Table 1:** The results of patient’s hormonal tests

**No.**	**Initials**	**Age [years]**	**17-OH-P [ng/ml]**	**Androstendion [ng/ml]**		**DHEAS [ng/ml]**		**TTE** **[ng/dl]**	**Estradiol [pmol/l]**	**FSH** **[IU/l]**	**LH** **[IU/l]**	**AMH** [ng/ml]*	**Inhibin B [pg/ml]**
1	MK	5.1	congenital adrenal hyperplasia excluded in neonatal period					<2.5 N: 2–25	-	0.83 N: 0.3–1.9	0.27 N: 0.1–0.4	>46	-
2	GH	6.2	congenital adrenal hyperplasia excluded in neonatal period					<2.5 N: 2–25	-	6.92 N:0,5–3.2	0,74 N: 0,1–0.4	130.6	-
3	WA	3.1	congenital adrenal hyperplasia excluded in neonatal period					<2.5 N: 2–25	<18.4 *N* < 18.4–73.4	36 N: 0.3–1.9	4,74 N: 0.1–0.4	1.51	5.9 N:25–325
4	TA	4.7	congenital adrenal hyperplasia excluded in neonatal period					<2.5 N: 2–25	-	2,9 N:0.3–1.9	0,44 N:0.1–0.4	-	-
5	KA	2.7 (all results at age 1.2)	congenital adrenal hyperplasia excluded in neonatal period					<2.5 N: 2–25	<18.4 *N* < 18.4–73.4	<170 N:0.5–5.5	28 N:0–4.1	0.03	3.4 N:25–325
6	NN	13.7 (all results at age	2.17		1.72		69.8 N:25.2–213.9	6.1 N:0–40	<18.35	2.74 N: 1.1–9	0.69 N:0.1–9.5	75.78	-
7	PO	2.7	0.32	0.45		<15 N: 0–15		<2.5 N: 2–10	<18.35	1.31 N:0.8–5.2	0.2 N: 0.1–0.3	87.5	299 N:25–325
8	SB	0.2	11.16 N:0.2–0.9	0.11		50.6 N:15–21.3		234 N: 75–400	48.02 N:18.4–73.4	3.67 N:0–5.5	5.16 N:0–4.1	73.0	283.4 N:25–325
9	NB	8.0	1.87 N:0.2–0.9	3.67		189.0 N: 25.2–213.9		<2.5 N:2–20	<18.35	9.62 N: 0.5–3.2	0.2 N:0.1–0.4	0.53	-
10	ZG	6.2	0.54 N:0.2–0.9	-		-		<2.5 N: 2–25	<18.4	0.5 N:0.3–1.9	0,16 N:0.1–0.4	6.24	-
11	KW	7.4	congenital adrenal hyperplasia excluded in neonatal period					<2.5 N:2–25	-	1.39 N:0.3–1.9	0.22 N:0.1–0.4	71.9	70 N:25–325
12	PK	4.2	0.83 N:0.2–0.9	0.06		<15 N;15–21.3		<2.5 N:2–25	<18.4	5.32 N:0.3–1.9	0.12 N:0.1–0.4	0.06	-
13	TJ	3.9	congenital adrenal hyperplasia excluded in neonatal period					<2.5 N:2–25 *at age 0.4 -234.7 N:1–177	<18.4	2.67 N:0.3–1.9 (at age −0.4)	7.87 N: 0–4.1 (at age −0.4)	9.08	-
14	ŚP	2.4	0.12 N:0.2–0.9	0.1		<15 N:15–80.1		<2.5 N:2–25	<18.4	31.55 N:0.1–0.7	0.9 N:0.1–0.2	0.15	-
15	GB	14.1	0.86 N:0.2–1.38	2.06		151 N:33.6–278.2		15.53 N:10–572	<18.4	N:134.0 1.2–7	35.4 N:0.8–6	-	-
16	GZ	17.9	0.41 N:follicular phase: 0.13 - 0.89 luteal phase: 0.16 - 2.05	3.07 N:follicular phase: 0.75–3.10, luteal phase: 0.94 - 3.20		103 N:111.8–363.3		24.01 8.4–48.1	49.69 (ERT)	66.1 N:1.6–9.8	60.9 N:1–39.3	-	-
17	OM	14.5	congenital adrenal hyperplasia excluded in neonatal period					30.1 N:5–40	178.0 (ERT)	4.04 N:1.6–9.8	0.88 N:1–39.3	-	-
18	RA	10.4	-	-		45.0 N: 33.6–278.2		<2.5 10–572	<18.4	146 N:1.2–7	27.9 N:0.8–6	<0.007	-
19	WM	5.8	-	-		-		<2.5 2–25	-	0.68 N: 0.3–1.9	0.23 N:0.1–0.4	-	-
20	WW	4.8	-	-		-		<2.5 2–25	-	0.55 N:0.3–1.9	0.21 N:0.1–0.4	-	-
21	WL	4.6 (all results at age 0.8)	0.73 N:0.2–0.9	0.29		<15 N:15–21.3		<2.5 N:2–7 Pregnyl test: (24 h) − 207.2, (72 h) −176.6	<18.35	10.8 N:0–5.5	0.93 N:0–4.1	>22.96	-
22	KF	10.3	-	-		105.0 N:33.6–278.2		<2.5 N:5–50	-	43.7 N:0.4–3.8	3.68 N:0.1–2.8	-	-
23	FB	12.1	1.31 N:follicular phase: 0.2–1.3; luteal phase: 1.0 - 4.5	0.87 N: follicular phase: 0.75–3.10, luteal phase: 0.94 - 3.20		45.0 N:25.2–213.9		<2.5 N:5–25	<18.35	120.0 N:1.1–9	27.3 N:0.1–9.5	0.05	<2,91 N:25,0 - 325,0
24	RM	4.4 (all results at age 1.5)	1.31 N:0.2–0.9	0.35		<15 N:15–21.3		<2.5 N:2–25	<18.35	0.6 N:0–5.5	0.25 N:0–4.1	-	-
25	JS	3.2 (results at age 0.8)	2.63 N:0.2–0.9	0.01		<15 15–21.3		<2.5 N:2–7	<18.35	1.13 N: 0–5.5	0.49 N:0–4.1	-	-
26	WD	2.8	<0.05 N:0.13–1.13	0.18		<15 N: 15–21.3		<2.5 N:2–25	<18.35	0.84 N:0.1–0.7	0.17 N: 0.1–0.2	138.9	<87,0 N: 25–325
27	KM	2.8	-	-		-		<2.5 N:2–25	<18.35	1.35 N: 0.3–1.9	<0.1 N: 0.1–0.4	>22.96 (at age 0.8)	159,7 N: 25–325 * at age 0.8
28	SF	5.2	congenital adrenal hyperplasia excluded in neonatal period					<2.5 N:2–25	<18.35	142.0 N:0–5.5	7.35 N: 0–4.1	<0.1	-
29	FJ	2.9	0.62 N:0.13–1.13	0.29		-		<2.5 N:2–25	<18.35	119.0 N:0–5.5	19.2 N:0–4.1	0.02	<4.0 N: 25–325
30	PI	8.2	0.09 N:0.13–1.13	0.44		-		<2.5 N:2–25	<18.35	2.73 N:0.3–1.9	0.13 N:0.1–0.4	0.17	-
31	KD	12.4	0.14 N:0.13–1.13	0.76		-		33.7 N:10–572	72 N: 41.4–159.0	1.65 N:1.2–7	1.99 N:0.8–6	84.0	204 N: 25–325
32	TP	12.5	1.38 N:0.2–0.9	2.02		137.0 N: 33.6–278.2		82.86	<18.35	1.52 N:0.4–3.8	0.6 N: 0.1–2.8	-	-
33	TJ	10.2	0.73 N:0.13–1.13	0.95		88.7 N:33.6–278.2		14.94 N: 5–50	<18.35	2.43 N: 0.4–3.8	0.12 N:0.1–2.8	104.0	-
34	KS	3.5 (all results at age 1.3)	1.2 0.13–1.13	<0.01		<15 15–21.3		<2.5 N:2–25 (0’, 48 h, 72 h in Pregnyl test); DHT < 17 pg/ml (0’, 48 h, 72 h in Pregnyl test)	<18.35	3.75 N:0–5.5	0.96 N:0–4.1	173.0	-
35	WT	2.1	0.73 N: 0.13–1.13	0.19		<15 N: 15–21.3		<2.5 N:2–25	<18.35	0.31 N:0.1–0.7	<0.1 N: 0.1–0.2	49.4	135.0 N:25–325
36	KR	2.9 (all results at age 0.3)	2.95 N: 0.13–1.13	0.45		23.4 N:15–21.3		<2.5 N:1–177	<18.35	<170.0 N: 0–5.5	57.1 N: 0–4.1	-	-
37	LM	17.8	-	-		219 N:91.8–512		36.24 N: 220–800	62.84 N: 41,4 - 159,0	63.3 N: 1.2–7	29.7 0.8–6	-	-
38	MW	6.1 (all results at age 0.8)	2.08 N:0.2–0.9	0.46		<15		<2.5 N: 2–7	-	0.72 N: 0–5.5	0.29 N: 0–4.1	18.2	20.5 N:25–325
39	KJ	12.4	0.73	1.73		168.0 N:33.6–278.2		214.0 N:5–50	50.0 N:41,4 - 159,0	1.13 N: 1.2–7	2.71 N:0.1–2.8	-	-

17-OH-P − 17-hydroxyprogesterone; DHEAS - dehydroepiandrosterone sulfate; TTE – testosterone; FSH - follicle-stimulating hormone; LH - luteinizing hormone; AMH - anti-Mullerian hormone; N- laboratory standard; (-) - no result obtained; ERT – estrogen replacement therapy; * the results were interpreted based on the article: Edelsztein NY,et al. Anti-Müllerian hormone as a marker of steroid and gonadotropin action in the testis of children and adolescents with disorders of the gonadal axis. Int J Pediatr Endocrinol. 2016;2016:20

Consent for genetic test was obtained from all patient’s parents and patients from 16 years old. Some of the patients’ parents also consented to report secondary findings in accordance with the American College of Medical Genetics (ACMG) guidelines [17] and to report carrier status of variants in selected genes associated with the occurrence of diseases with recessive inheritance, severe course and early onset.

### Whole-exome sequencing (WES)

Samples of blood were collected on filter paper (dried blood spot). The obtained material was analyzed by whole-exome analysis using NGS, including identification of single nucleotide variants (SNVs), insertions, deletions (InDels), Copy Number Variations (CNVs), uniparental disomies (UPDs), and mitochondrial DNA (mtDNA) variants (with heteroplasmy ≥ 15%). Study included coverage of the entire exome (~20,000 genes), +/- 10 bp exon-intron boundaries. A depth of coverage of 20× was considered and a report was generated indicating the proportion of each gene covered to this level.

The final report from laboratory included the primary results (with descriptions), potentially significant results (with descriptions) and secondary – in case of obtained consent. Variants were classified following the ACMG and genomics recommendations [18]. The report included variants of classes 3–5 (pathogenic, potentially pathogenic and VUS). Sanger sequencing was performed to confirm SNVs. In case of CNV, Array Comparative Genomic Hybridization (aCGH) or Multiplex Ligation-dependent Probe Amplification (MLPA) were used for confirmation.

After receiving the results, genotype-phenotype correlation was performed again and additional predictive programs assessing the pathogenicity of variants were used - Franklin by QIAGEN – see: links under bibliography [1] and GeneBe [2]. Classification of pathogenicity was also compared with data from ClinVar [3].

In patients with molecularly confirmed diagnosis and in patients with presence of VUS in genes, variant segregation in the family was recommended and appropriate tests were ordered (results are not included in this publication).

## Results

In 8 children genetic causes of DSD phenotype were detected (Table 2).

**Table 2:** Characteristics of 46,XY DSD patients with confirmed molecular diagnosis

	age [years]	Sex assigned at birth/sex of rearing/gender identity [F/M]]#	External / internal genital phenotype/clinical assessment	Hormonal tests	Gene	Variant	Diagnosis; OMIM; ORPHA	Classification
MK	5.1	M	genital tubercle, scrotal hypospadias, labio-scrotal folds, gonads in the labio-scrotal folds no Müllerian structures visible in ultrasound	prepubertal FSH, LH, TTE concentration	*AR*	c.2567 G > A	**Partial Androgen Insensitivity Syndrome (PAIS); 312,300; 90,797**	pathogenic
GH	6.2	F	external female genitalia, history of right inguinal hernia repair, bilateral abdominal testicles, no Müllerian structures visible during laparoscopy	status after gonadectomy - high levels of LH, FSH	*AR*	c.2301del	**Complete Androgen Insensitivity Syndrome (CAIS); 300,068; 99,429**	
WA	3.1	M	genital tubercle, urethral meatus at the tip of the genital tubercle, bilateral impalpable testicles, completely fused labial-scrotal folds, laparoscopy – bilateral hypoplastic testicles with split epididymides and spermatic ducts, no visible Müllerian structures	hypergonadotrophic hypogonadism	*DHX37*	c.2020C > T	**46,XY sex reversal 11; 273,250; 983**	
TA	4.7	M	micropenis, urethral meatus at the tip of micropenis, scrotal hypoplasia, bilateral cryptorchidism, ultrasound - testicles	hypergonadotrophic hypogonadism	*AR*	c.2134C > G	**Partial Androgen Insensitivity Syndrome (PAIS); 312,300; 90,797**	potentially pathogenic
KA	2.7	M	genital tubercle/micropenis, urethral meatus at the tip of micropenis, labioscrotal folds, ultrasound and laparoscopy - bilateral testicular atrophy,	hypergonadotrophic hypogonadism, no increase in testosterone level in pregnyl test	*AR*	c.1792A > G	**Partial Androgen Insensitivity Syndrome (PAIS); 312,300; 90,797**	
NN	13.7	F	external female genitalia, inguinal hernia, bilateral palpable gonads; ultrasound – testicles visible in the inguinal canals, MRI - no uterus/Müllerian structures visible, hist-path - male gonad	prepubertalconcentration of estradiol, TTE, LH, FSH in the norm, in the pregnyl test - increase in TTE concentration	*AR*	c.2375C > T	**Complete Androgen Insensitivity Syndrome (CAIS); 300,068;** **99429**	
PO	2.7	F	external female genitalia, inguinal hernia, ultrasound – testicles visible in inguinal canals, no visible Müllerian structures	high (for female) TTE level, prepubertal FSH, LH, estradiol levels	*AR*	c.2375C > T	**Complete Androgen Insensitivity Syndrome (CAIS); 300,068;** **99429**	
SB	0.2	M	micropenis, scrotal hypospadias, split scrotum, testicles palpable in the scrotum, ultrasound – testicles	FSH, LH, TTE, AMH concentrations within the range for a male child during mini-puberty	*HSD17B3*	c.277+4A > T c.133C > T	**17-beta hydroxysteroid dehydrogenase III deficiency; 264,300; 752**	
NB	8.0	F/M (transgender boy)	genital tubercle, the urethral opening at the base of the genital tubercle, labioscrotal folds, gonads in labioscrotal folds, ultrasound – gonads suggestive as testicles, no ovaries and uterus	prepubertal concentration of FSH, LH, TTE and estradiol, low level of AMH	*NR5A1*	c. 11_12del	**46,XY sex reversal 3; 612,965; 251,510**	VUS
ZG	6.2	M	genital tubercle, urethral opening at the top of the genital tubercle, scrotum, bilateral cryptorchidism – testicles palpable in the groin, ultrasound - testicles	prepubertal concentration of FSH, LH, TTE, low level of AMH	*DHX37*	c.2598_2600delGTAinsATG	**46,XY sex reversal 11; 273,250; 983**	
KW	7.4	M	micropenis, urethral meatus at the top of micropenis, hypoplastic scrotum slightly fused, left testicle in the scrotum, right testicle palpable in the groin, ultrasound - testicles	prepubertal concentration FSH, LH, TTE	*AR*	c.721A > G	**Partial Androgen Insensitivity Syndrome (PAIS); 312,300; 90,797**	
PK	4.2	M	penis, urethral meatus at the top of penis, asymmetric scrotum, left-sided cryptorchidism, small right gonad palpable in the scrotum, ultrasound – small right gonad, laparoscopy - hypoplasia of the right testicle, hypoplastic testicular vessels on the right side	hypergonadotrophic hypogonadism, low level of AMH	*MAMLD1*	c.834C > A	**Hypospadias 2, X-linked; 300,758; 95,706**	
TJ	3.9	M	penis, scrotal hypospadias, labioscrotal folds, impalpable left testicle, right gonad in labioscrotal folds, laparoscopy – left dysgenetic gonad with accompanying Müllerian structures- hist-path of right gonad -testicular tissue	hypergonadotrophic hypogonadism, AMH within the range	*SOS2*	c.586 G > C	**Noonan syndrome 9; 616,559; 648**	
ŚP	2.4	M	genital tubercle, urethral meatus at the tip of the genital tubercle, fused labioscrotal folds, bilateral cryptorchidism, ultrasound - testicles	hypergonadotrophic hypogonadism, low level of AMH	*FAM111A*	c.1660 G > C	**Kenny-Caffey syndrome, type 2; 127,000; 2333**	
GB	14.1	M	penis, urethral meatus at the tip of the glans, scrotum, impalpable testicles, laparoscopy - bilateral atrophic testicles	hypergonadotrophic hypogonadism	*DHX37*	c.1516 G > A	**46,XY sex reversal 11; 273,250; 983**	

*Legende: # According to Polish law, a child’s gender must be chosen as male or female; there is no option for a “third gender” or an unspecified gender. Therefore, in many cases, parents are assisted in making a gender decision by a panel consisting of a pediatric endocrinologist, a geneticist, a surgeon, a psychologist, and a lawyer. AMH - anti-Mullerian hormone, FSH - follicle-stimulating hormone, LH - luteinizing hormone, TTE- testosterone*

**Fig. 1:**
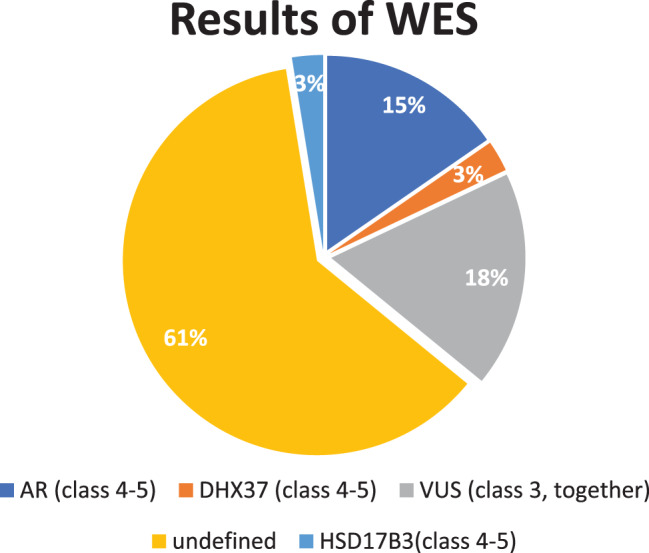
Percentage of patients with or without pathogenic variants in genes correlated with 46,XY DSD

**Fig. 2:**
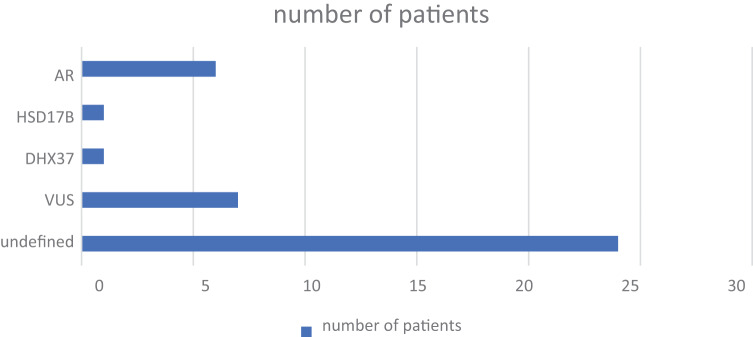
Number of patients with or without pathogenic variants in genes correlated with 46, XY DSD

A definitive diagnosis, based on the detection of a class 4–5 variant and correlating with the patient’s phenotype, was confirmed in 8/39 (21%) of patients; variants were classified as pathogenic (in 3 patients) - including *AR* and *DHX37* genes, and potentially pathogenic (in 5 patients) − 4 in the *AR* gene and 1 in *HSD17B3.* Patient with compound heterozygote in *HSD17B3* is awaiting for parent’s verification (2%). In case of 7/39 (18%) patients we found VUS, including *NR5A1, DHX37, AR, MAMLD1, SOS2, FAM111A* genes; in these cases family-based variant segregation analyses and further case reanalysis are necessary to confirm or exclude suspected genes as connected with DSD (currently in progress). VUS are presented in the Table 1 with characteristics of patients phenotype. No variants that could be related to DSD symptoms were found in the remaining children. The pie chart presents the percentage of patients with a diagnosis, taking into account variants in the tested genes (Figs. 1 and 2).

Additionally, the analysis also revealed pathogenic variants in other genes, not related to DSD. The detailed description is included in the Table 3.

**Table 3:** Phenotype and molecular diagnosis of concomittant diseases in 46,XY DSD patients and results of assessment of the occurrence of pathogenic variants according to ACMG

Patients	Age of diagnosis [years]	sex assigned at bith/sex of rearing [F/M]]	phenotype	Gene	variant	Diagnosis’ OMIM; ORPHA	classification
OM	14.5	F	psychomotor delay, motor stereotypies, autism features	56 genes	1q21.1 duplication	1q21.1 duplication syndrome; 612,475; 250,994	pathogenic
ZG	6.2	M	dilation of the ascending aorta	*NOTCH1*	c.5785 G > T	type 1 aortic valve defect; 109,730; 402,075	potentially pathogenic
TP	12.5	M	intellectual disability	*HUWE1*	c.7433C > T	X-linked intellectual disability; 309,590; 85,328	VUS
TJ (TP’s brother)	10.2	M	intellectual disability	*a)HUWE1* b) 110 genes (72 protein-coding genes)	a) c.7433C > T b) 3q24q25.33 deletion	a) X-linked intellectual disability; 309,590; 85,328 b) associated with developmental delays, dysmorphic features, autism, and microcephaly; -; 262,019	a)VUS b)pathogenic
WT	2.1	M	hipercholesterolemia	*LDLR*	DUP:chr19-11215896-11222312	hypercholesterolemia familial, 1; 143,890; 391,665	potentially pathogenic
NB	8.0	M (transgender boy)	no symptoms	*GJB2*	c.101T > C homozygous	hearing loss, autosomal recessive, 1A; 220,290; 90,636	pathogenic
KJ	12.4	M	no symptoms	*PKP2*	c.2014-1 G > C	arrhythmogenic right ventricular dysplasia, familial, 9; 609,040; 217,656	pathogenic

Phenotype of DSD patients for whom the diagnosis could not be established at the molecular level is presented in Table 4.

**Table 4:** Phenotype of DSD in 46,XY patients without molecular diagnosis

Patients	age [years]	assigned sex/ sex of rearing[F/M]	phenotype	hormonal tests	ORPHA
GZ	17.9	F	as a newborn - genital tubercle, opening of the genitourinary sinus on the perineum, labioscrotal folds, gonads in labioscrotal folds, ultrasound - in the projection of the right adnexa a solid tumor, history of feminizing genitoplasty at age of 4 and bilateral inguinal hernia repair at age of 5; at the age of 13: short (4 cm) vaginal dimple, persistent clitoromegaly, right gonadal tumour visible in ultrasound; laparoscopy and hist- path - right side - testicular tissue, with Leydig cell hyperplasia, gynandroblastoma, left side - ovary without follicles, with fallopian tube, testicular rete and epididymis, laparoscopy and hit-path – dysgerminoma (?)	status post gonadectomy – high levels of FSH, LH, undetectable estradiol, during hormone replacement therapy	325345 363582
OM	14.5	F	external female genitalia, MRI - uterine hypoplasia, absent left gonad, small right ovary; laparoscopy - Müllerian structures - small uterus, fallopian tubes, small dysgenetic gonads, gonadectomy performed - hist-path – dysgenetic gonads	status post gonadectomy – high levels of FSH, LH, undetectable estradiol, during hormone replacement therapy	251510
RA	10.4	M	genital tubercle, urethral opening at the top of the genital tubercle, labioscrotal folds, impalpable gonads, ultrasound - testicles in abdomen, MRI - gonadal dysgenesis	hypergonadotropic hypogonadism, low level of AMH	251510
WM	5.8	M	bent penis/genital tubercle, penoscrotal hypospadias, scrotum, gonads in the scrotum, ultrasound - testicles	prepubertal FSH, LH, TTE concentrations	95706
WW	4.8	M	penis, urethral meatus at the base genital tubercle, penoscrotal hypospadias, scrotum, gonads in the scrotum, ultrasound - testicles	prepubertal FSH, LH, TTE concentrations	95706
WL	4.6	M	micropenis, urethral opening at the tip of the genital tubercle, hypoplastic scrotum, asymmetrical, impalpable gonads, ultrasound - left gonad in abdomen (testis?), missing right gonad, Müllerian structures on the right side, laparoscopy – left side - testis, removal of Müllerian structures	low testosterone level during “mini-puberty”, in the test with pregnyl, normal testosterone secretion	251510 983
KF	10.3	M	penis, urethral opening at the tip of the glans, hypoplastic scrotum, impalpable gonads in the scrotum, ultrasound - small right testis, left gonad absence, laparoscopy - removal of the atrophic right testis and implantation of a prosthesis, testicular atrophy	hypergonadotropic hypogonadism	251510983
FB	12.1	F	external female genitalia, ultarasound – no visible gonads, bilateral gonadectomy (germinoma), MRI after gonadectomy- uterus, single tissue band in the vagina;	status post gonadectomy – high levels of FSH, LH, undetectable estradiol	242 363582
RM	4.4	M	penis, penile hypospadias, scrotal transposition, testicles in scrotum	prepubertal FSH, LH, TTE concentrations	95706
JS	3.2	M	bent penis, perineal hypospadias, bifid scrotum, impalpable gonad right inguinal hernia, ultrasound - testicles	prepubertal FSH, LH, TTE concentrations	95706
WD	2.8	M	bent penis scrotal hypospadias, bifid scrotum, testicles in scrotum	prepubertal FSH, LH, TTE concentrations	95706
KM	2.8	M	bent penis, urethral meatus at the border of the labioscrotal folds, fused labioscrotal folds, gonads in the inguinal canals, ultrasound – testicles, persistent Müllerian ducts	prepubertal FSH, LH, TTE concentrations, in the pregnyle test - normal testosterone levels	251510
SF	5.2	M	penis, urethral meatus at the tip of glans, scrotum, impalpable gonads in the scrotum, laparoscopy - right atrophic testicles, left testicular hypotrophia	hypergonadotropic hypogonadism	251510 983
FJ	2.8	M	penis, urethral opening at the tip of the glans, scrotum, impalpable gonads in the scrotum, laparoscopy - agenesis of the right testicle and hypoplastic left testicle	hypergonadotropic hypogonadism	251510 983
PI	8.2	M	penis, urethral opening at the tip of the glans, hypoplastic scrotum, impalpable left gonad in the scrotum, laparoscopy - left testicular atrophia	prepubertal FSH, LH, TTE concentrations	251510 983
KD	12.4	M	micropenis, urethral meatus at the tip of micropenis, scrotum, palpable testicles in scrotum	FSH, LH, TTE within the normal range (TTE in lower limit),	325357
TP	12.5	M	micropenis, urethral meatus at the tip of micropenis, bifid, poorly developed scrotum, testicles palpable in scrotum	FSH, LH, TTE concentrations within the normal range	325357
TJ	10.2	M	micropenis, perineal hypospadias, labioscrotal folds, left testicle palpable high in the groin, right impalpable gonad	FSH, LH, TTE concentrations within the normal range	95706 983
KS	3.5	M	the genital tubercle hidden in the scrotal-labial folds, the scrotal-labial folds fused, the right testicle palpable in scrotal-labial folds, impalpable left gonad	low testosterone level during “mini-puberty”, in the pregnyle test - low testosterone and dihydrotestosterone levels	251510 983
WT	2.1	M	penis, urethral meatus at the tip of penis, scrotum, right impalpable gonad, left testcle in the scrotum, laparoscopy - right testicular agenesia with visible right fallopian tube / mullerian structure/	prepubertal FSH, LH, TTE concentrations,	251510 983
KR	2.9	M	penis, urethral meatus at the tip, scrotum, impalpable gonads, laparoscopy – bilateral testicular atrophy	hypergonadotropic hypogonadism	251510 983
LM	17.8	M	penis, urethral meatus at the tip, scrotum, impalpable gonads, laparoscopy bilateral testicular atrophy	hypergonadotropic hypogonadism	251510 983
MW	6.1	M	genital tubercle, urethral meatus at the tip, labio-scrotal folds, right gonad palpable high in the groin, impalpable left gonad	low testosterone level during “mini-puberty”, in the pregnyle test - low testosterone levels	251510 983
KJ	12.4	M	penis, urethral meatus at the tip, scrotum, right impalpable testicle, laparoscopy – right testicular atrophy	FSH, LH, TTE concentrations within the range	983

*Legende:. AMH - anti-Mullerian hormone, FSH - follicle-stimulating hormone, LH - luteinizing hormone, TTE- testosterone*

VUSs connected with another disorders than DSD and not corresponding with patient phenotype are not included in this article.

## Discussion

Diagnosis of DSD demands comprehensive approach. Taking a medical history, physical examination and assessment of genitals are the first steps. Further diagnostic includes hormone measurements, imaging, and genetic diagnostic. The routine use of genetic testing for reaching a diagnosis is increasingly playing an important role in the diagnostic process. Various techniques are currently used in genetic diagnostics [19]. Conventional karyotyping is frequently a first choice during diagnostic process of DSD. We decided to perform WES as second step of genetic diagnosis as this method is high-throughput screening technology, which allow to analyze and identify the presence of many variants at the same time [20]. Additionally, the advantage of WES is the possibility of identification of new DSD-related genes. However, as mentioned, even this method does not allow for diagnosis in more in about half of the cases. Based on our studies we have a similar observation as genetic cause was not determined in 63% of patients. Importantly, the detection rate for genetic causes of DSD 46,XY has not changed much in the last decade [4, 5, 14, 21, 22]. It is possible that additional genes associated with DSD will be identified in the future using WES, and it also appears that mutations in intronic sequences, i.e. non-coding DNA, may be responsible for some of these cases. The fact that 63% of patients remained without a molecular diagnosis, and a further 18% carried VUS, suggests that many pathogenic mechanisms may lie outside exonic regions or involve complex genomic alterations that WES cannot capture. Such changes could be investigated in future studies using whole-genome sequencing (WGS). Emerging evidence demonstrates that WGS offers a significant diagnostic advantage over WES in a wide spectrum of rare developmental conditions, including DSD. While WES interrogates only the protein-coding 1–2% of the genome, WGS provides comprehensive coverage of non-coding regions, enhancers, promoters, intronic splice-altering variants, untranslated regions, mitochondrial DNA, repetitive elements and complex structural variants. Large clinical studies show that WGS increases diagnostic yield by an additional 10–20% beyond WES, primarily through detection of non-coding or structural variants [23, 24]. In DSD specifically, WGS has revealed pathogenic enhancer disruptions and rearrangements involving genes such as *SOX9, SRY* and *MAP3K1*—findings undetectable by WES [25]. These observations strongly support WGS as the next-tier testing strategy in unresolved 46,XY DSD.

Androgen insensitivity syndrome (AIS) is the most frequent etiology of 46,XY DSD patients [6]. Affected individuals present a broad spectrum of under-virilization. Three phenotypes of AIS were distinguished: complete (typically female external genitalia; CAIS), partial (a wide spectrum of undervirilizied external genitalia; PAIS) or mild (male external genitalia, gynecomastia and/or infertility; MAIS) [26]. Similarly, in our study the percentage of AIS diagnoses was the highest - we recognized 3 PAIS as well as 3 CAIS cases; patient KA (Table 1) with diagnosis of PAIS has variant for which conflicting data are reported. Additionally, one patient (KW, Table 1) has VUS in *AR* gene - diagnosis not established. Especially in these both situations, verification of detected variants should be performed in mother of patients (disease X-linked recessive) and re-evaluation of pathogenicity of each variant should be performed based on medical databases after some time. In literature, 800 variants in *AR* gene were reported in AIS patients. *AR* variants are identified in 90–95% of CAIS, but only in 28–50% of PAIS [27]. Thus, diagnostic remains demanding.

In literature, pathogenic and potentially pathogenic variants in *DHX37* were reported in patients with 46,XY DSD with frequency of 14%. Taking into consideration embryonic testicular regression syndrome (ETRS) phenotype (atypical genitalia, micropenis, absence of testicular tissue) this frequency increases according to some authors to 50% [28]. In our study, we confirmed the presence of a pathogenic variant in the *DHX37* gene in one patient, while VUS in this gene were described in two other patients. It is necessary to analyze the segregation of the variant within the family and track information appearing in medical databases about these variants (possible change to a variant of a class 4–5 and then making a diagnosis).

46,XY DSD due to 17β-HSD-3 deficiency is a disorder that consists of a defect in the last phase of steroidogenesis when androstenedione is converted to testosterone and estrone to estradiol. Patients present female-like or atypical genitalia at birth. Most affected newborns can be raised as females [29]. Change to male gender role behavior at puberty has been frequently described in patients who were raised as female [30]. In a review of all adult patients reared as female and not castrated during childhood, 61% kept the female gender, while 39% identified with male gender [29]. The disorder is due to homozygous or compound heterozygous variants in the *HSD17B3* gene. As reported, in our patient we found 2 variants (no.8 in Table 1)– first was classified as pathogenic, while classification of the second was ambiguous (class 3–4). Based on NGS we cannot be sure if variants are in *cis* or in *trans* position. Therefore, we verified parents using Sanger sequencing and confirmed in *trans* position of above mentioned variants.

*NR5A1* (*SF1* in the past) is involved in the development of the urogenital ridge. NR5A1 protein is essential for gonadal and adrenal formation [31]. Kohler et al. analyzed *NR5A*1 gene 46,XY patients with severe underandrogenization and identified heterozygous mutations in 18.5% patients. Thus they concluded that *NR5A1* mutations are a relatively frequent cause of 46,XY DSD [32]. Lin et al. (2007) revealed mutations that showed loss of function in adrenal, Leydig, and Sertoli cells lines [33]. *NR5A1* pathogenic variants have been associated with a wide range of phenotypes including female phenotype with isolated partial and complete gonadal dysgenesis, 46,XY undervirilization, vanishing testes, male infertility and adrenal insufficiency as a rare finding [34]. The variant described in our patient has not been previously described in the literature and is classified as a VUS (our case report is in press). Due to general knowledge about pathogenic variants in this gene we included in patient’s recommendations regular monitoring for adrenal insufficiency.

The remaining DSD-associated variants detected in our study and classified as VUS included *SOS2, FAM111A*, and *MAMLD1* genes. Some of the patients’ symptoms overlap with those associated with pathogenic variants in these genes. However, a diagnosis has not been established; they remain under observation and parents are during investigation.

The considerable number of VUS identified in our cohort highlights a major limitation of current genomic diagnostics in 46,XY DSD. A VUS cannot be classified as benign, nor can it be reliably dismissed as unrelated to the phenotype. Instead, VUS require a structured, dynamic clinical approach that includes: (1) thorough phenotype–genotype correlation; (2) family segregation studies; (3) assessment with multiple in-silico prediction tools; (4) re-evaluation according to updated ACMG/AMP criteria; and (5) continued clinical monitoring, especially during puberty. Importantly, several VUS detected in our study affect genes with strong biological relevance to gonadal development, including *NR5A1, DHX37* and *AR*. These variants are plausible candidates for the underlying etiology and should not be interpreted as ‘negative results’. As functional genomics continues to advance, many VUS may eventually be reclassified as pathogenic or likely pathogenic. Therefore, patients carrying VUS require ongoing multidisciplinary follow-up, particularly regarding endocrine function, potential adrenal insufficiency (*NR5A1*), gonadal tumor surveillance, and psychological care.

Additionally to diagnostic of DSD, some patients were diagnosed with pathogenic/potentially pathogenic variants in genes unrelated to sex development. Some of them were asymptomatic, such as a patient homozygous for the pathogenic variant in *GJB2* gene, which is correlated with hearing loss; a patient diagnosed with a variant reported according to the ACMG recommendation and associated with arrhythmogenic right ventricular dysplasia familial, 9 – sent to further cardiological diagnostic; or patient with hypercholesterolemia detected during hospitalization in our department that have found duplication of a fragment of the *LDLR* gene. In one patient with dilation of the ascending aorta we recognized type 1 aortic valve defect. X-linked intellectual disability was supposed in 2 brothers. It seems, therefore, that the choice of the WES method allowed us to make several diagnoses that we did not expect.

Thanks to our study it was possible to make a diagnosis and further therapeutic decisions in some patients. Gender incongruence are common in this group of patients, and it is impossible to clearly predict which gender a patient identifies with based on the results of a genetic test. However, it is worth noting that in the case of CAIS, almost all patients identified as women, while in case of PAIS gender identity can be both, female or male. In pathogenic variants in *DHX37* we can observe male, female, male to female identification, in case of *17HSDB3* deficiency most patients keep the female social sex; some change to male social sex [35]. Higher prevalence of addictions and suicidal thoughts or suicide attempt in 46,XY DSD than those observed in the general population, demand special approach [36], especially in case of *17HSDB3* deficiency with high percentage of gender identity disorder [37]. Gender-affirming treatment, psychological care for patients with gender dysphoria is necessary for their mental health. As mentioned, in Poland a child’s gender must be chosen as male or female; there is no option for an unspecified gender. It is very important to remember that gender identification may be different than that assigned at birth and children as well as their parents should be under care of psychologist and in some case, child psychiatrist. A conversation between the attending physician and the school counselor may also prove valuable if the parents so wish.

Results of genetic test are also helpful in preventive cancer screenings or the timing of the gonadectomy. It is important that any disturbance in the gonadal development increases the risk of developing gonadal malignancies. Overall, neoplastic transformation of germ cells in dysgenetic gonads occurs in 20–30% of 46,XY DSD individuals, but the risk varies among 46,XY DSD etiologies. Germ cell tumor risk is lowest (<5%) in patients with defects in androgene action or synthesis (CAIS), whereas the highest risk (15–60%) is observed in 46,XY gonadal dysgenesis [38]. In CAIS Sertoli cell adenomas, seminomas and malignant sex-cord stromal tumor were described. Prediction malignancy risk is inconclusive - risk of malignancy in gonads is estimated at 1.3% in CAIS individuals before puberty, but it can reach about 15–30% in adults patients [39–43]. Thus, gonadectomy may be delayed until puberty is complete. In case of PAIS, Wooster et.al described brothers with penoscrotal hypospadias who developed infiltrating ductal cancers of the breast at ages 75 and 55 years [44]. Rarely germ-cell neoplasia in situ on histology is described in patients with *DHX37* mutations. Thus, careful monitoring of the patient, as well as decisions regarding possible gonadectomy, should be determined individually by a multidisciplinary consultation.

Most of 46,XY DSD patients face infertility due to atypical gonadal development, endocrine disturbances, anatomical changes or prophylactic gonadectomy. Patient with CAIS, PAIS, mutations in *HSD17B3, DHX37* are infertile (OMIM). However, fertility preservation technologies have been improved in recent years, so successful biological fertility was obtained in a man with PAIS after high-dose testosterone therapy followed by intracytoplasmic sperm injection [45]. In individuals with a 46,XY karyotype and pathogenic variants of the *NR5A1* gene, the phenotype may be variable (OMIM), but the chances of preserving fertility appear to be very low. Results of genetic test can be also helpful in prediction about potential fertility based on assisted fertility and preservation techniques.

Genetic testing is also often very important for parents. Further testing of parents using the Sanger method allows for the identification of possible carriers of abnormal variants in the family and genetic counseling to determine the likelihood of recurrence of the disease in subsequent pregnancies.

It is worth mentioning that high-throughput genetic testing such as WES, requires significant bioinformatic resources. Additionaly, newly discovered variants are not always clearly classified. Currently, many predictive tools are used to classify variants, but their pathogenicity assessments are not always consistent. Similarly, the ClinVar database does not always provide a clear answer regarding the significance of a given variant. Knowledge is constantly evolving, but just as important as making a diagnosis is, it is equally crucial not to make diagnosis when there is no clear evidence suggesting a link between a found variant and a disease entity.

WES is currently the method that has an advantage over NGS panels, however, only 1–2% of genome is covered in this diagnostic method), so many 46,XY DSD cases still elude precise genetic classification, necessitating comprehensive clinical evaluation and multidisciplinary approaches. Despite the modest diagnostic yield of 21% in our cohort, the clinical impact of WES was substantial. WES enabled identification of pathogenic or likely pathogenic variants that directly informed decisions regarding sex assignment counselling, monitoring for gonadal malignancy, surgical timing, and fertility considerations. Furthermore, WES revealed additional pathogenic variants unrelated to DSD in several patients, allowing early detection or prevention of cardiovascular, metabolic or neurodevelopmental conditions. These findings demonstrate that WES provides significantly broader clinical benefit than targeted gene sequencing alone.

It is important to verify the status of the identified variants, especially VUS in the biological parents of the patients. In addition, it is recommended to re-analyze WES about 12–24 months due to the continuous progress of knowledge in the field of genetics. Due to this re-analysis it may be possible to determine the genetic cause of DSD in those patients in whom WES examination has not yet allowed for a diagnosis. WGS remains a thing of the future.

## Conclusions

In conclusion, whole-exome sequencing enabled the identification of pathogenic or likely pathogenic variants in a subset of well-phenotyped patients with 46,XY DSD and provided clinically relevant information extending beyond DSD-associated genes. Although the overall diagnostic yield was modest, the clinical utility of WES was substantial, guiding decisions regarding sex assignment, surveillance for gonadal malignancy, endocrine management and family counselling. A considerable number of patients carried variants of uncertain significance, many in genes with established roles in sex development. These variants cannot be dismissed as non-causal and require longitudinal re-evaluation, segregation analysis and careful clinical follow-up. The high proportion of unresolved cases highlights the limitations of WES in detecting non-coding, regulatory and structural variants. Therefore, whole-genome sequencing represents a necessary next step to improve diagnostic yield in 46,XY DSD, offering comprehensive genomic coverage and the potential to clarify the underlying aetiology in patients who remain without a definitive molecular diagnosis.

## Data Availability

The results are stored in the computer memory. Data are available on request. To obtain access to data, please contact the author for correspondence.

## Abbreviations

ACMG: American College of Medical Genetics
aCGH: Array comparative genomic hybridization
AIS: Androgen insensitivity syndrome
CAIS: Complete Androgen Insensitivity Syndrome
CNV: Copy number variation
DSD: Differences in sex development
NGS: Next-generation sequencing
PAIS: Partial Androgen Insensitivity Syndrome
WES: Whole-exome sequencing
